# Complete Traumatic Rupture of the Pancreas by a Horse Saddle: A Case Report

**DOI:** 10.7759/cureus.52570

**Published:** 2024-01-19

**Authors:** Pablo Avila-Sanchez, Javier A Pliego-Zermeño, Natalia M Barron-Cervantes, Carlos Chan

**Affiliations:** 1 Hepato-Pancreato-Biliary Surgery, Instituto Nacional de Ciencias Medicas y Nutrición Salvador Zubiran, Mexico City, MEX; 2 General Surgery, American British Cowdray Medical Center, Mexico City, MEX; 3 School of Medicine, Universidad Panamericana, Mexico City, MEX; 4 Hepato-Pancreato-Biliary Surgery, Instituto Nacional de Ciencias Medicas y Nutricion Salvador Zubiran, Mexico City, MEX

**Keywords:** pediatric emergency room, distal pancreatectomy, horse-riding abdominal trauma, pediatric trauma, pancreatic trauma

## Abstract

Pancreatic trauma is one of the least observed diagnoses in the emergency room, much less in pediatric emergencies. Any cause of direct abdominal blunt trauma can cause it. With only a few cases presented in the literature, horse accidents have been associated with this complication, but it has been never seen in literature as a case where the horse-riding saddle is the one causing the pancreatic trauma, until now. Emphasizing the importance of an early diagnosis is the key point, but more importantly, to highlight that the correct diagnostic approach will grant the opportunity for a lesion in the main pancreatic duct to be identified, which will allow a timely surgical approach, increasing overall survival rates and decreasing morbidity in these patients. Here lies the importance of not only utilizing a specific study, such as a computerized tomography (CT) scan to evaluate abdominal trauma but also using other image studies that are better suited for pediatric patients, such as magnetic resonance image (MRI) with cholangiopancreatography (MRCP).

## Introduction

Pancreatic trauma is an uncommon complication presented with abdominal trauma, with a reported incidence of 3.7%-11% [1]. Its significance lies in the potential for morbidity, with 23%-53% of all patients presenting additional complications, such as pancreatic insufficiency, type 3 diabetes, and chronic diarrhea. The mortality rate associated with this complication is 17%-70%, mainly due to pancreatic fistula, pancreatitis, hemorrhage, abscesses, pseudocysts, and associated vascular injuries [2]. A delay of six to 12 hours is estimated to considerably increase morbidity, emphasizing the importance of early identification in the outcomes of these patients [3]. Management in pediatric patients is generally conservative, although, in cases when the main pancreatic duct (PD) is compromised, early surgical treatment with resection and drainage is recommended [4]. This case is presented in order to further expand the knowledge about pancreatic trauma kinetics, and early diagnosis and show our proposal for surgical management, allowing other doctors to analyze the diagnostic and therapeutic approach. We present the case of a 13-year-old male patient who presented to the emergency room with abdominal blunt trauma caused by the pommel of a horse-riding saddle in a first-level private surgical center in Mexico City.

## Case presentation

**Figure 1:**
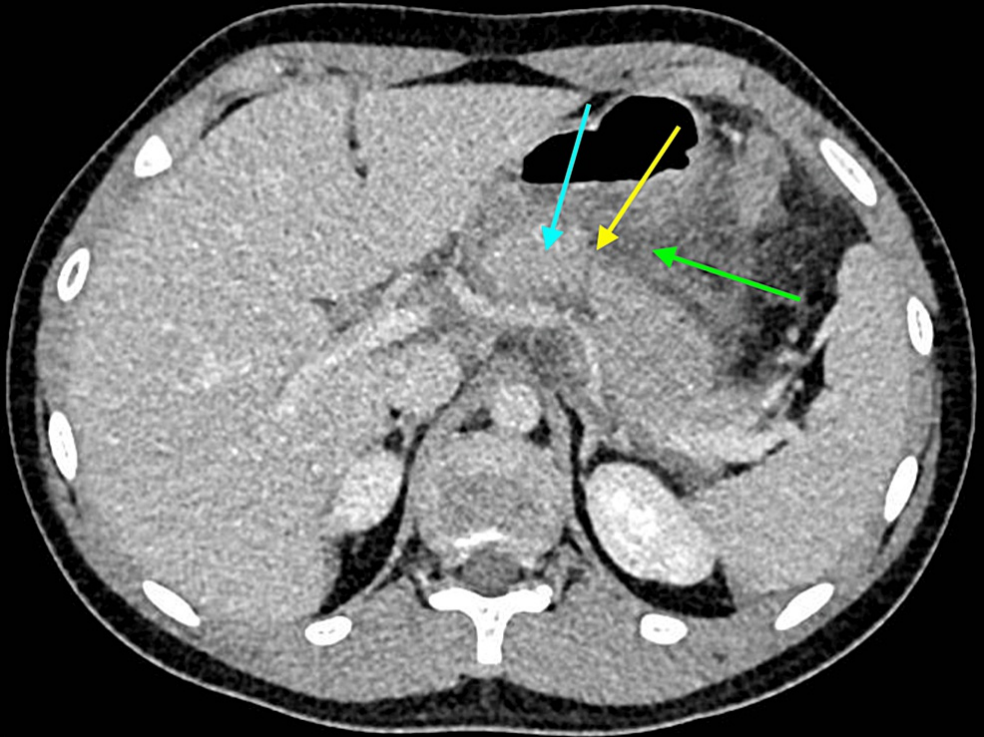
Abdominal CT scan with intravenous contrast. Diffuse inflammation of the pancreas (blue arrow) with the presence of a peripancreatic fluid collection (green arrow). Possible rupture of the pancreatic parenchyma (yellow arrow).

A 13-year-old male patient with no past medical history presented to the emergency department with severe abdominal pain following blunt trauma to the epigastrium from the pommel of a horse-riding saddle while getting up from the ground after falling while riding a horse. After the event, he presented nausea and vomiting, which was initially associated with intense pain. Upon admission, he was hemodynamically stable. Upon physical examination, a 7-cm ecchymosis in the epigastric region was presented, but no signs of peritoneal irritation were found. During his stay at the emergency room laboratories were requested, which showed elevated serum levels of amylase (1,556 U/L) and lipase (2,899 U/L) and leukocytosis (13.5 x 10^3^ U/L), everything else within normal limits. Adequate fluid resuscitation and analgesia with Acetaminophen 1 gram IV and Tramadol 50 mg IV were initiated. CT scan revealed diffuse inflammation of the pancreas with the presence of a peripancreatic fluid collection that did not allow the pancreas to be completely visualized, possible rupture of the pancreatic parenchyma at the level of the body was observed (Figure 1).

**Figure 2:**
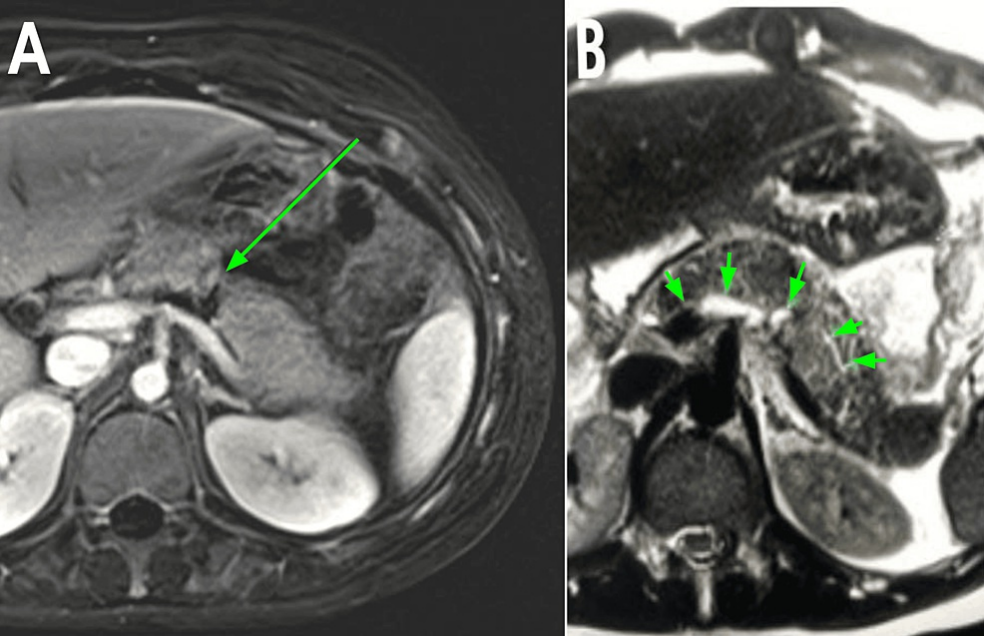
Magnetic resonance image (MRI) with cholangiopancreatography (MRCP). (A) Transection of the entire thickness of the pancreatic parenchyma at the level of the pancreatic body (green arrow). (B) Discontinuity of the PD (green arrows).

Due to the uncertainty of imaging diagnosis, a magnetic resonance image (MRI) with cholangiopancreatography (MRCP) was ordered, which reported a disconnected PD with complete rupture of the pancreatic parenchymal body, corresponding to a Grade III laceration according to the American Association for the Surgery of Trauma (AAST) classification (Figures 2A, 2B). Pediatric surgery was consulted afterwards and a hepatopancreatobiliary surgeon was called upon.

**Figure 3:**
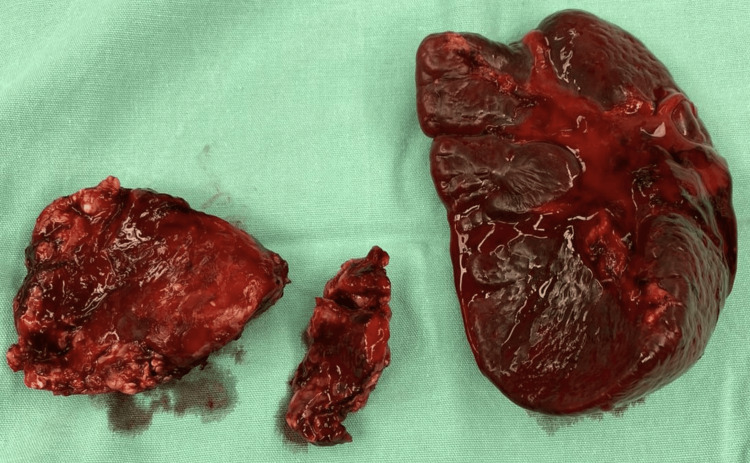
Surgical specimen. Distal pancreatectomy with splenectomy.

After a careful review of the case and images, an exploratory laparotomy was planned and performed. With the patient in the operating room, a jugular central catheter was placed as well as a nasogastric tube. A midline incision was performed and upon entering the cavity hemoperitoneum and omentum fat saponification, splenic laceration, and a peripancreatic fluid collection containing pancreatic content in the omental bursa were reported. After drainage of blood clots and fluid in the omental bursa a complete longitudinal rupture at the level of the pancreatic body with a bloody proximal edge was identified. Distal pancreatectomy with splenectomy using a surgical stapler was done (Figure 3).

The closure of the pancreatic stump was adequate, and a closed suction drainage was placed in the splenic bed because of the magnitude of the pancreatic trauma and the high risk of postoperative pancreatic fistula (POPF). The rest of the surgical exploration showed no associated injuries. Total surgical bleeding was calculated at 350 mL and surgical time was 90 minutes. The patient was extubated in the operating room and translated to the intensive care unit (ICU) for continuous monitoring. Throughout his stay analgesia with Acetaminophen 1 gram intravenous (IV) every 12 hours and Tramadol 25 mg IV every 24 hours were administered, also he started antibiotic therapy with Ertapenem 1 gram IV every 24 hours for five days. After 24 hours, he was discharged from the ICU, and a liquid diet was initiated without complications. After 72 hours, he was still asymptomatic, on oral intake, and within normal post-op evolution when the abdominal drainage amylase was measured with a result of 101,190 U/L therefore a grade A POPF, also known as biochemical fistula or not clinically relevant POPF (NCR-POPF), was diagnosed based on 2016 ISGPF classification [5]. As the drainage output was progressively decreasing, he was discharged from the hospital on the eight postoperative days. Two weeks later, he went to his follow-up appointment where he was evaluated clinically and biochemically. He presented completely asymptomatic and no alterations were presented in his control laboratories. Also, no drainage output was presented so it was removed with no further complications.

## Discussion

Pancreatic trauma is one of the most uncommon complications presented with abdominal blunt trauma, the most common cause reported is motor vehicle accidents and bicycle-related incidents. However, other causes have been described in the literature, even horse accidents. The few cases reported were associated with direct kicking to the abdomen by horses, and none of them describe trauma caused by the riding chair, as far as we know, this makes our case the first describing this trauma mechanism in literature [6,7]. When speaking about trauma kinetics, we believed the direct impact on the epigastrium by the saddle pommel caused compression of the pancreas against the vertebral bodies, resulting in complete organ rupture. This confirms theories previously mentioned in literature where it is proposed that overall, this is the result of a high-energy trauma [7]. The importance of this lies in the fact that despite being protected by professional riding equipment, it does not exclude pancreatic trauma as a possible complication in a fall, even without evidence of direct horse contact with the patient.

**Figure 4:**
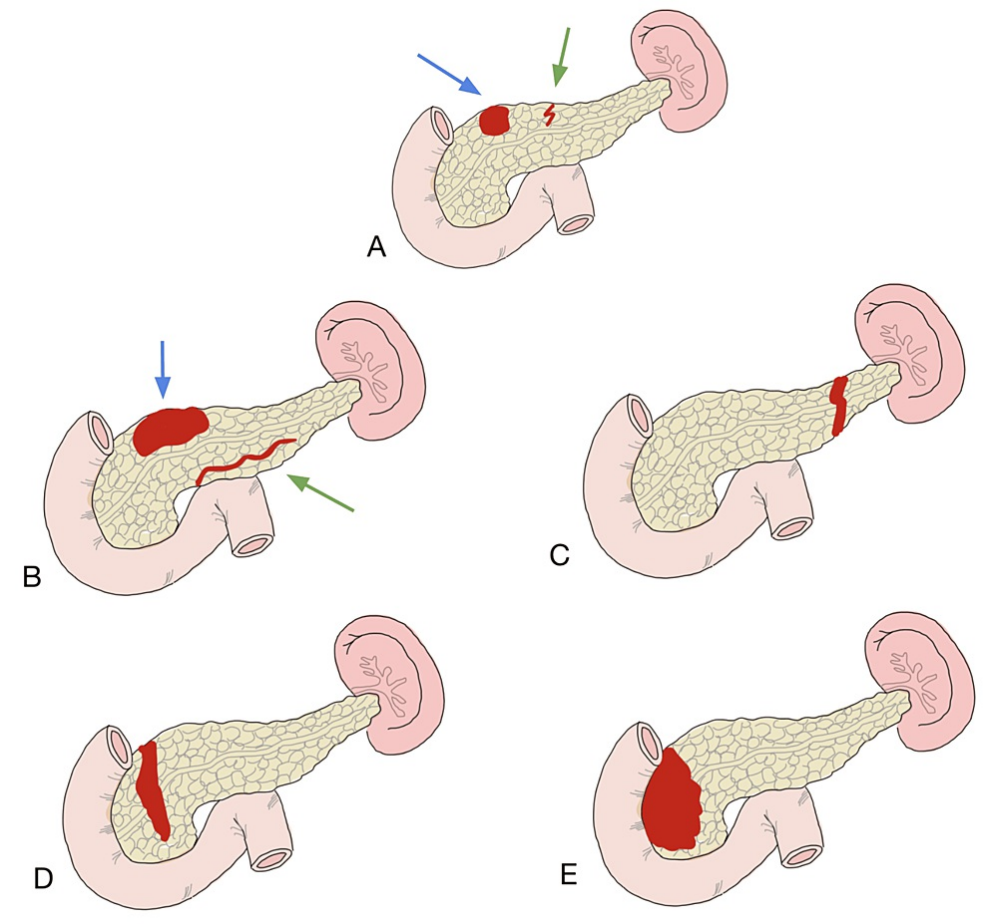
AAST classification of pancreatic trauma. (A) Grade I: hematoma with minor contusion without ductal injury (blue arrow) or superficial laceration without any ductal injury (green arrow). (B) Grade II: hematoma with major contusion without ductal injury or tissue loss (blue arrow) or major laceration without any ductal injury or tissue loss (green arrow). (C) Grade III: distal transection or pancreatic parenchymal loss. (D) Grade IV: proximal transection or pancreatic parenchymal loss involving the ampulla. (E) Grade V: massive disruption of the pancreatic head. Image Credits: Natalia M. Barron-Cervantes, adapted from [4]. AAST - American Association for the Surgery of Trauma

When speaking about an early diagnostic approach CT scan is usually the initial imaging study due to its excellent specificity, nevertheless, its limited sensitivity restricts its utility. Additionally, in pediatric patients, its diagnostic yield has been shown to be low, making MRCP the preferred imaging modality in these cases. This image study is useful for assessing PD injuries by demonstrating the PD directly or by showing secondary changes, such as signal intensity difference in pancreatic parenchyma and caliber difference in the PD, even when the rupture is not as clear as in this case [8]. Correct evaluation of the PD involvement and anatomical correction is critical to determine its classification according to the AAST (Figures 4A-4E) and its possible therapeutic approach, either conservative or surgical.

Conservative management includes close monitoring in the ICU, no oral feeding, analgesia, antibiotics, and percutaneous drainage if needed. Some studies published in recent years propose the use of Somatostatin or Sandostatin (chemical analog) is indicated in cases where the pancreatic fistula is presented with clinical manifestations (fever, leukocytosis, abdominal pain, and anorexia) or an output of more than 500 mL per day; however, the evidence is still very controversial, and it is not recommended in a standardized way. Therefore, conservative management is indicated in patients with grade I and grade II injuries according to the AAST classification. On the other hand, surgical management is indicated in grade III, grade IV, and grade V injuries. There is still a lot of debate regarding the best surgical strategy; however, most bibliographies can agree on the fact that pancreatic trauma management should always be individualized based on the injury extension, location, and associated trauma. Many surgical processes can be done, including stent placement into the PD, distal pancreatectomy, total pancreatectomy and even only performing damage control surgery [9].

Damage control surgery is the best alternative for severe life-threatening cases where severe acute inflammation makes safe resection impossible, in this case endoscopic stent placement is also recommended. Nevertheless, pancreatic resection surgery has been proven to be one of the best surgical procedures as it has been associated with less need for reoperation, so it should be considered over drainage [8]. When speaking about pancreatic resection, a distal pancreatectomy with splenectomy is one of the most commonly performed procedures for distal injuries, but proximal injuries require a stage-specific approach usually executed by an HPB surgeon [10]. In this particular case, it is important to mention that the primary surgeon involved in the case is an HPB specialist who decided that as part of this surgery, he was going to place closed suction drainage in the splenic bed as the probability of presenting a POPF was high due to the extension and magnitude of the pancreatic injury. This drainage allowed the NCR-POPF to be identified and controlled, leading to no further treatment or intervention being needed, minimizing the probability of getting a secondary complication associated with another intervention.

## Conclusions

The integrity of the PD and the delay in diagnosis are key determinants of morbidity and mortality in pancreatic trauma. Therefore, a thorough inquiry into the trauma's kinetics and early MRCP were crucial in this case to characterize the complete rupture of the duct and guide appropriate surgical management. The trauma mechanism involved in this case is one of a kind and it emphasizes the importance of considering it in the differential diagnosis even though the patient was protected by professional equipment and the horse did not hit him directly. While some authors have reported successful outcomes with resection and reconstruction using pancreaticojejunostomy, in this patient, the decision was made for resection alone due to the presence of bloody pancreatic tissue, which hindered a proper anastomosis, demonstrating that the best surgical approach is always individualized to the case. Another very important thing to highlight about this case is that having a surgeon specialized in HPB surgery allowed us to properly determine the risk of POPF and placing the drainage in the surgery eventually allowed us to manage this complication less invasively, minimizing the morbidity and mortality of our patient.
